# Epidemiological characterization of *Clonorchis sinensis* infection in humans and freshwater fish in Guangxi, China

**DOI:** 10.1186/s12879-022-07244-2

**Published:** 2022-03-18

**Authors:** Yihong Xie, Kaushi S. T. Kanankege, Zhihua Jiang, Shun Liu, Yichao Yang, Xiaoling Wan, Andres M. Perez

**Affiliations:** 1 Department of Epidemiology and Biostatistics, School of Public Health, Guangxi Medical University, 22 Shuangyong Road, P.O. Box 530021, Nanning, Guangxi China; 2 Department of Veterinary Population Medicine, College of Veterinary Medicine, University of Minnesota, P.O. Box 55414, Minneapolis, USA; 3 Division of Parasitic Disease Prevention and Control, Guangxi Zhuang Autonomous Region Center for Disease Prevention and Control, P.O. Box 530021, Nanning, Guangxi China

**Keywords:** Clonorchiasis, *Clonorchis sinensis*, Epidemiology, Determinants

## Abstract

**Background:**

Clonorchiasis is a widespread yet neglected foodborne disease with over 85% of all cases found in China. Guangxi province, located in southeastern China, ranks among the highest endemic provinces. We explore the epidemiological status and determinants of *Clonorchis sinensis* (*C. sinensis*) infection in humans and freshwater fish in Guangxi, China.

**Methods:**

Data on *C. sinensis* infection in humans from January 2008 to December 2017were extracted from the China Information System for Disease Control and Prevention. An active surveillance of *C. sinensis* infection in fish was conducted in 2016–2017. County level data including potential environmental, social-economical and behavioral determinants was also collected. Univariate and multivariate logistic regression models were used to explore the determinants of *C. sinensis* infection in humans and fish. Simple and multiple zero-inflated Poisson regression models were fit to assess the associated factors of clonorchiasis in humans at the county level.

**Results:**

Totally, 4526 *C. sinensis* cases were reported between 2008 and 2017, with an annual prevalencerate of 0.96/100,000 persons. Of 101 counties in Guangxi, 97 reported at least 1 case. Among 2,098 fish samples, 203 (9.7%) from 70 counties contained *C. sinensis*. The rate was higher in small fish including *Pseudorasbora parva* (45.3%), *Misgurnus anguillicaudatus* (41.2%), *Hemicculter leuciclus* (34.5%), unclassified small fishes (30.9%), *Cyprinidae* (20.0%)*, Cirrhinus molitorella* (16.4%), *Carassius auratus* (13.6%) and *Cyprinus carpio* (13.3%), while it was lower in fish species that are usually used in preparing raw fish dishes including *Ctenopharyngodon idellus* (3.6%)*, Spinibarbus denticulatus* (3.7%)*, Monopterus albus* (6.4%)*, Cyprinus carpio* (4.4%), *Oreochromis mossambicus* (3.3%) and *Spualiobarbus Curriculus* (6.6%). The *C. sinensis* infection in fish was only associated with fish species. The estimated human clonorchiasis prevalence at the county level was positively associated with raw fish consumption habits and certain rivers.

**Conclusions:**

Clonorchiasis is highly prevalent in both humans and freshwater fish in Guangxi. Environmental, social-economic and behavioral determinants contribute to the high prevalence as well as the significant differential distribution by county. Regular surveillance should be implemented for clonorchiasis to demonstrate the change in epidemiology and burden, which will benefit the design of interventions.

**Supplementary Information:**

The online version contains supplementary material available at 10.1186/s12879-022-07244-2.

## Background

Clonorchiasis is an important yet neglected foodborne parasitic disease in East Asian countries, including China, South Korea, Vietnam and parts of Russia. The disease is caused by a trematode known as *Clonorchis sinensis* (*C. sinensis*) [1–3]. A high morbidity can occur including disorders and carcinogenesis in the liver and biliary system [1, 4]. Approximately 15 million people are infected with *C. sinensis* globally, of which > 85% are distributed across China [2, 5]. Based on two national surveys, the major endemic areas in China are concentrated in four provinces, including Guangxi and Guangdong in the southeast, and Heilongjiang and Jilin in the northeast [6, 7]. In Guangxi, the infection rate of *C. sinensis* increased from 1.4% in 1990 to 4.0% in 2003 [7, 8], while the number of counties with *C. sinensis* cases expanded from 23 in 1990 to 59 in 2003 [8] and 96 in 2017 based on surveillance data from the China Information System for Disease Control and Prevention. Guangxi ranks second in China in terms of prevalence [7, 8]. Thus, clonorchiasis causes a significant health burden in Guangxi [9].

*C. sinensis* infection in humans was found to be significantly related to the infection of metacercariae in freshwater fish and the habit of ingesting raw fish [1]. More than 100 species of freshwater fish have been documented in the transmission of *C. sinensis* [10]. The rate of *C. sinensis* in fish is not homogeneous, but varies across geographical regions and by fish species [8, 11–15]. Usually, studies focus only on one aspect, either on the human infection or on the parasitic infection in freshwater fish. Limited research has explored the association between them spatially. Furthermore, it is of value to capture environmental, social-economic, and behavioral determinants in both humans and freshwater fish. However, such information is limited, especially in Guangxi [16]. This study aimed to capture the epidemiology of *C. sinensis* infection in both humans and freshwater fish at the county level in Guangxi and to determine epidemiologically important determinants. Results of this study are expected to guide the implementation of interventions.

## Methods

### Study area

Guangxi is located in southern China at latitude of 20.54 N to 26.24 N and longitude of 104.26 E to 112.04 E, covering a total area of 236,700 km^2^ with a population of 48.85 million in 2017. The province consisted of 14 prefectural-level administration divisions, which include 101 counties.

### C. sinensis infection in humans

Data of *C. sinensis* infection in humans from January 2008 to December 2017 was extracted from the China Information System for Disease Control and Prevention. This is an online, real-time surveillance system in China. As Guangxi is one of the four endemic areas in China, *C. sinensis* cases are requested to report into this system within 24 h after diagnosis in Guangxi. The definition of *C. sinensis* was made according to the national diagnostic criteria for clonorchiasis (WS309-2009) [17]. Those who have of epidemiology history of eating raw or undercooked freshwater fish, or live in/come from epidemic areas, and with the presence of *C. sinensis* eggs in the stool by using modified Kato's thick-smear technique were diagnosis as *C. sinensis* infection.

### Collection and detection of freshwater fish

An active surveillance program was conducted by the Guangxi Center for Disease Prevention and Control (Guangxi CDC) to monitor *C. sinensis* infection in freshwater fish from April 2016 to December 2017. The sampling protocol followed the National Food Chemical Pollutants and Hazardous Factors Monitoring Manual issued by the Chinese National Center for Food Safety Risk Assessment and the Food Safety Risk Monitoring Manual issued by the Guangxi Health Bureau. In brief, a minimum of 20 to 25 freshwater fish samples were collected from each county, with a minimum weight of 500 g in each sample. Samples were collected from at least 2 to 3 local markets, restaurants or fish ponds, and < 5 samples were collected from any single place. The fish included three types, namely live fish, fresh chilled fish, raw fish dishes (at least accounting for 20–30% of the total samples). The species of fish included the species that were frequently consumed locally such as *Ctenopharyngodon idellus* and *Oreochromis mossambicus* and those that were usually infected with *C. sinensis* such as *Cyprinus carpio* [11] and *Pseudorasbora parva* [12, 13]. The sampling was repeated twice in each county in the study period, one between April and June and another from July to September.

Fish samples were transferred at 4℃ to Guangxi CDC and processed for laboratory testing within 48 h. An artificial digestion technique was applied for detection of metacercariae following the guideline entitled ‘Identification for metacercaria of *C. sinensis* in fish’ (SN/T 2975-2011). The metacercariae were differentiated based on morphological characteristics under microscope. The metacercariae is round or oval in shape, measures about (121–150) μm * (85–140) μm, it contains two layers of walls. The oral and ventral suckers along with excretory bladder are present on the larva inside [18]. The concurrent trematode metacercariae infection of *Metorchis orientalis* was also examined in the fish samples.

### Collection of environmental, social-economical and behavioral information

Meteorological data in 2016 at the county level were obtained from the Guangxi Meteorological Bureau, including mean evaporation (mm), sunshine hours (h), relative humidity (%), barometric pressure (pha), rainfall (mm), temperature (℃), highest temperature (℃), and lowest temperature (℃) [19]. The per capita GDP (yuan) in 2016 was extracted from the Guangxi Statistical Yearbook [20]. The river basins system in Guangxi was categorized based on the Guangxi Water Conservancy Information System [21].

A questionnaire survey was implemented to capture practices in ingesting raw freshwater fish. A minimum of two to three local health officers or CDC staff were enrolled from each county. Participants were asked whether local people prepared raw freshwater fish at home and whether local restaurants provided raw freshwater fish dishes. Correspondingly, each county was categorized into one of four types: type 1 (i.e. no consumption of raw fish) indicated the county where local people did not eat raw fish at home and raw fish dishes were not available at local restaurants; type 2 (seldom consumption) where local people did not eat raw fish at home but raw fish dishes were available from local restaurants; type 3 (sometimes consumption) where some local communities ate raw fish at home and raw fish dishes were available in local restaurants; type 4 (frequent consumption) where the local people ate raw fish at home frequently and raw fish dishes were available at local restaurants and markets.

### Data analysis

Data management and analysis was performed using R version 4.0.2 (R core team Vienna, Austria, 2018). The ‘epicalc’ [22], ‘pscl’ [23, 24], and ‘spatialreg’ packages [25] were used in the analysis. Descriptive statistics (e.g. rates and proportions) were used to describe *C. sinensis* infection in humans and in freshwater fish by geography and time. Two separate regression models were fit to analyze the infection in freshwater fish (logistic regression) and humans (zero inflated Poisson regression). Univariate and multivariate logistic regression models were used to assess associated factors of *C. sinensis* infection in freshwater fish, including fish species, sample status, and sampling place. The associations and the relative goodness of fit of the full and final models were evaluated using the P-values from the likelihood ratio test and Akaike information criteria (AIC) values, respectively [26]. Thematic maps were used to depict the spatial distribution of *C. sinensis* infection in humans and freshwater fish using Quantum GIS. Standardized data, considering the number of reported (observed) and expected (based on population size) number of human clonorchiasis cases by county, were mapped using GeoDa [27].

Considering a big number of countries have zeros case reported in each year and the under reported nature of human clonorchiasis [3, 5], a simple and multiple zero-inflated Poisson (ZIP) regression model was fit to assess the associated factors hypothesized to influence disease risk at the county level to reduce biased estimate if the zeros are ignored for the sake of simplifying the analysis [28, 29]. A simple inflation model where all zero counts were assumed to have the same probability of belonging to the zero component was specified. The number of reported cases of human clonorchiasis was the dependent variable. Population at risk was used as the offset. The independent variables included the positive rate of *C. sinensis* in freshwater fish, raw fish consumption habits, rivers, areas based on a pre-existing categorization of foodborne pathogens in Guangxi [30], meteorological factors and social economic factors including gross domestic product (GDP), which were collected at the county level. The goodness-of-fit of the full and best-fitted models was assessed using AIC values. The simple and multiple ZIP regression results were presented as exponentiated values and were interpreted as ratios. The residuals of the finalized ZIP model was subjected to *Moran’s I* global spatial autocorrelation test to determine the presence of unexplained spatial autocorrelation.

## Results

### *C. sinensis* infection in human

**Fig. 1:**
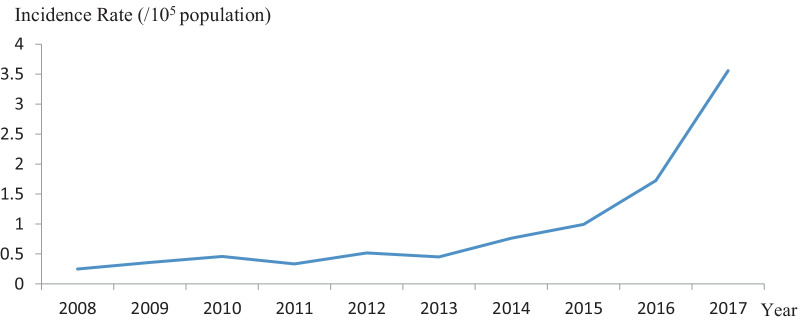
Prevalence of human clonorchiasis in Guangxi, China from 2008 to 2017

**Fig. 2:**
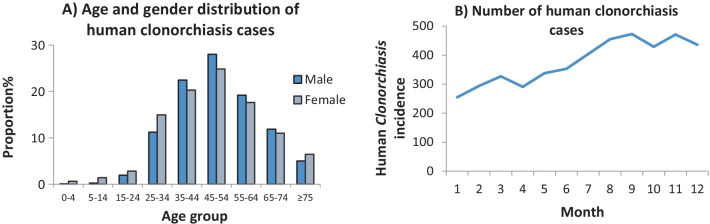
Distribution of 4526 clonorchiasis cases stratified by age and gender (Panel **A**) and monthly trend over time (Panel **B**) in Guangxi, China, 2008 to 2017

**Fig. 3:**
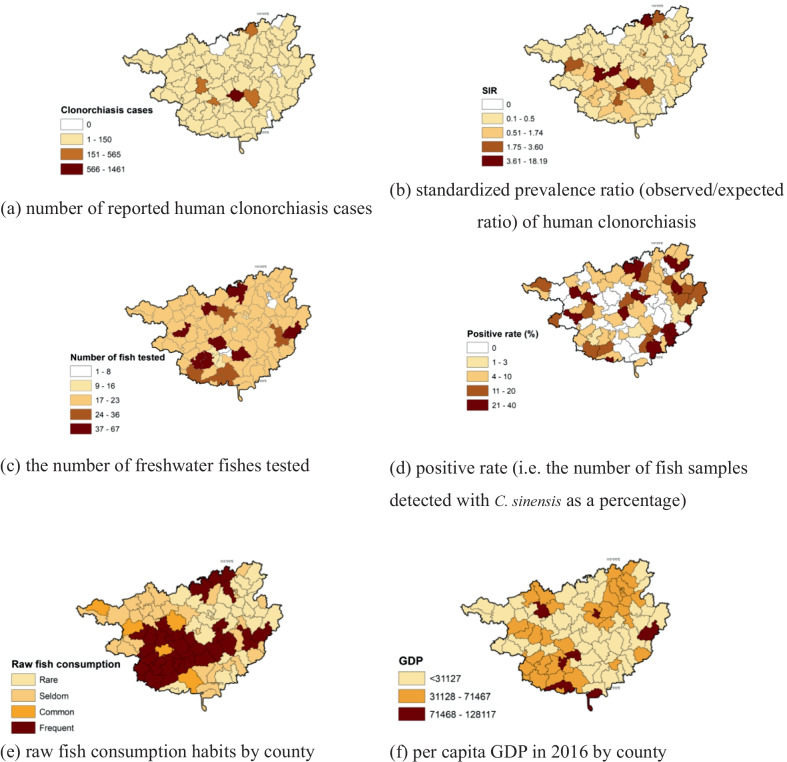
Human clonorchiasis cases reported between 2008 and 2018 (**a**, **b**), surveillance results of fishes (**c**, **d**), raw fish consumption habits (**e**), and the per capita GDP (**f**) at the county level, Guangxi, China. We drew this figure using Quantum GIS software

Totally, 4526 *C. sinensis* cases were reported between 2008 and 2017, with an annual prevalence rate of 0.96/100,000. No fatality was reported. The prevalence rate increased significantly from 2008 to 2017 (Fig. 1) (P-value for trend < 0.05), which was increase from 0.2 in 2008 to 3.6 per 100,000 populations in 2017. The number of reported cases in males (3,890) was 5.2 times higher than that in females (626). Most of the reported cases (97.9%) were adults aged over 20 years, the proportion of age group of 25–34, 35–44, 45–54, 55–64, 65–74 years were accounted for 11.7%, 22.1%, 27.6%, 19.0% and 11.7%, respectively. The youngest with an infection was a 2-year-old male and a 3-year-old female. A high number of cases were reported during July and December, which account for 58.9% of the total cases (Fig. 2). Most cases (58.2%) were farmers, followed by the retirees (9.9%), housewives (7.1%), officers (6.3%), and worker (3.1%). All but four counties reported at least 1 case (Fig. 3a). The main epidemic areas were distributed around Nanning city (the capital of Guangxi), as well as counties in the western, eastern, and northern parts (Fig. 3a, b).

### C. sinensis infection in freshwater fish

Totally, 2098 freshwater fish samples comprising 61 fish species were collected from all 101 counties (Fig. 3c). These included 25 specific fish species and 36 unclassified small fish species, of which only 1–5 samples were collected from each species, so they were grouped as “unclassified small fishes” in the analysis. Among them, 203 fish samples were detected with *C. sinensis*, with a total positive rate of 9.7%. 5 fish samples were concurrent infection by *Metorchis orientalis*. *C. sinensis* metacercariae were detected from 70/101 counties (69.3%) (Fig. 3d).

For each fish species, *Pseudorasbora parva* and *Misgurnus anguillicaudatus* showed the highest infection rate (45.3% and 41.2%, respectively), followed by *Hemicculter leuciclus* (34.5%), unclassified small fishes (30.9%), *Cyprinidae* (20.0%)*, Cirrhinus molitorella* (16.4%), *Carassius auratus* (13.6%), *Cyprinus carpio* (13.3%), *Monopterus albus* (9.1%), *Pelteobagrus fulvidraco* (7.7%) and *Aristichthys nobilis* (7.1%). The infection rate of *Siniperca chuatsi* and *Katsuwonus pelamis* were 11.1% (1/9) and 10% (1/10), respectively. The positive rate was relatively low in the following six fish species that are often used in preparing raw fish dishes, including *Ctenopharyngodon idellus* (3.6%)*, Spinibarbus denticulatus* (3.7%)*, Monopterus albus* (6.4%)*, Cyprinus carpio* (4.4%), *Oreochromis mossambicus* (3.3%) and *Spualiobarbus Curriculus* (6.6%) (Table 1).

**Table 1:** Positive rate of *C. sinensis* in freshwater fish and logistic regression results identifying risk factors of *C. sinensis* infection in freshwater fish in Guangxi, China from 2016 to 2017

Variables	Test No.	Positive No. (positive rate %)	Univariate analysis		Multivariate analysis	
			Crude OR (95% CI)	*P-value*	Adjusted OR (95% CI)	*P-value*
Fish status						
Raw fish dishes	528	21 (4.0)	Reference	–	Reference	–
Live fish	1323	149 (11.3)	3.06 (1.92–4.90)	< 0.001	1.58 (0.88–2.82)	0.122
Fresh chilled fish	247	33 (13.4)	3.72 (2.11–6.58)	< 0.001	1.45 (0.74–2.84)	0.279
Sampling place						
Restaurant	389	17 (4.4)	Reference	–	Reference	-
Farm/wholesale	90	9 (10.0)	2.43 (1.05–5.65)	0.039	1.22 (0.46–3.22)	0.691
Market	1619	177 (10.9)	2.69 (1.61–4.48)	< 0.001	1.21 (0.65–2.23)	0.548
Fish species						
*Ctenopharyngodon idellus*	416	15 (3.6)	Reference	–	Reference	–
*Monopterus albus*	109	7 (6.4)	1.83 (0.72–4.61)	0.198	1.76 (0.70–4.45)	0.230
*Parabramis pekinensis*	23	1 (4.3)	1.21 (0.15–9.62)	0.854	0.93 (0.12–7.44)	0.943
*Spualiobarbus Curriculus*	76	5 (6.6)	1.88 (0.66–5.34)	0.235	1.77 (0.62–5.07)	0.285
*Colossoma brach ypomum*	25	1 (4.0)	1.11 (0.14–8.79)	0.918	0.85 (0.11–8.83)	0.882
*Siniperca chuatsi*	9	1 (11.1)	3.34 (0.39–28.45)	0.270	2.84 (0.33–24.45)	0.342
*Cyprinus carpio*	30	4 (13.3)	4.11 (1.27–13.28)	0.018	3.18 (0.96–10.51)	0.058
*Pelteobagrus fulvidraco*	39	3 (7.7)	2.23 (0.62–8.06)	0.222	1.69 (0.46–6.26)	0.430
*Carassius auratus*	132	18 (13.6)	4.22 (2.06–8.64)	< 0.001	3.28 (1.55–6.95)	0.002
*Katsuwonus pelamis*	10	1 (10.0)	2.97 (0.35–24.98)	0.316	2.87 (0.34–24.53)	0.335
*Hemicculter leuciclus*	58	20 (34.5)	14.07 (6.66–29.71)	< 0.001	11.45 (5.26–24.91)	< 0.001
*Cyprinidae*	85	17(20.0)	6.68 (3.19–14.01)	< 0.001	5.30 (2.45–11.47)	< 0.001
*Cyprinus carpio*	251	11 (4.4)	1.23 (0.55–2.71)	0.616	1.02 (0.45–2.30)	0.961
*Hypophthalmichthys molitrix*	56	2 (3.6)	0.99 (0.22–4.45)	0.990	0.80 (0.17–3.62)	0.767
*Cirrhinus molitorella*	67	11 (16.4)	5.25 (2.30–12.00)	< 0.001	4.30 (1.84–10.08)	< 0.001
*Lateolabrax japonicus*	24	1 (4.2)	1.16 (0.15–9.19)	0.887	0.96 (0.12–7.67)	0.969
*Oreochromis mossambicus*	246	8 (3.3)	0.90 (0.38–2.15)	< 0.001	0.74 (0.30–1.80)	0.507
*Pseudorasbora parva*	64	29 (45.3)	22.15 (10.86–45.17)	< 0.001	17.07 (8.07–36.08)	< 0.001
*Misgurnus anguillicaudatus*	17	7 (41.2)	18.71 (6.26–55.93)	< 0.001	14.37 (4.67–44.28)	< 0.001
*Silurus asotus*	59	1 (1.7)	0.46 (0.06–3.55)	0.457	0.36 (0.05–2.82)	0.332
*Aristichthys nobilis*	56	4 (7.1)	2.06 (0.66–6.43)	0.215	1.69 (0.53–5.35)	0.375
*Spinibarbus denticulatus*	82	3 (3.7)	1.02 (0.29–3.59)	0.981	1.13 (0.32–4.01)	0.854
*Monopterus albus*	33	3 (9.1)	2.67 (0.73–9.75)	0.163	2.06 (0.55–7.66)	0.282
*Clarias fuscus*	29	1 (3.4)	0.95 (0.12–7.49)	0.1363	0.74 (0.09–5.86)	0.773
*Acipenser sinensis*^a^	8	0 (0.0)	–	–	–	–
Unclassified small fishes^b^	94	29 (30.9)	11.93 (6.07–23.45)	< 0.001	9.28 (4.55–18.93)	< 0.001
Total	2098	203 (9.7)	–	–	–	–

^a^*Acipenser sinensis* was not included in the analysis as the number of positive fish was 0

^b^Including 36 unclassified small fish species with only 1–5 samples for each species

Among 528 samples of raw fish dishes, the major fish species were *Ctenopharyngodon idellus* (42.0%, 222/528), *Spinibarbus denticulatus* (11.2%, 59/528), *Monopterus albus* (9.7%, 51/528), *Cyprinus carpio* (8.9%, 47/528), *Oreochromis mossambicus* (8.3%, 44/528) and *Spualiobarbus Curriculus* (6.6%, 35/528), which accounted for 86.7% (458/528) of all samples. However, fish species that were highly infested were not commonly used in raw fish dishes, including *hemicculter leuciclus*, unclassified small fishes, *Cyprinidae, Cirrhinus molitorella*, *Carassius auratus* and *Cyprinus carpio* (6.6%, 35/528). Only 0.9% (5/528) of raw fish dishes was made using unclassified small fishes, and no raw fish dishes were made using *Pseudorasbora parva*, *Misgurnus anguillicaudatus*, *Pelteobagrus fulvidraco*, *Parabramis pekinensis* or *Colossoma brach ypomum*. Among the 528 raw fish dishes sampled, approximately 4% were detected with *C. sinensis*.

Univariate and multivariate results exploring factors associated with *C. sinensis* infection in freshwater fish are shown in Table 1. Fish status, sampling place, and species were all significantly associated with *C. sinensis* infection in the univariate analysis. However, only fish species remained significant in the multivariate analysis. To explore the risk of consuming different fish species, the most commonly consumed fish species (*Ctenopharyngodon idellus)* was set as the reference group. Compared to this fish, *Pseudorasbora parva*, *Misgurnus anguillicaudatus*, *Hemicculter leuciclus*, unclassified small fishes, *Cyprinidae*, *Cirrhinus molitorella* and *Carassius auratus* species had higher rates of infection with adjusted odds ratios (OR) and 95% confidence intervals (CI) of 17.07 (8.07–36.08), 14.37 (4.67–44.28), 11.45 (5.26–24.91), 9.28 (4.55–18.93), 5.30 (2.45–11.47), 4.30 (1.84–10.08) and 3.28 (1.55–6.95), respectively.

### Factors associated with human infection

Raw fish consumption was common in central counties located around the capital city of Nanning and some areas in the west-north belt of Guangxi (Fig. 3e). Among 101 counties, the practice of consuming raw fish dishes was classified as none (32.7%), seldom (5%), sometimes (32.7%), and frequent (29.7%) consumption based on the survey results.

**Fig. 4:**
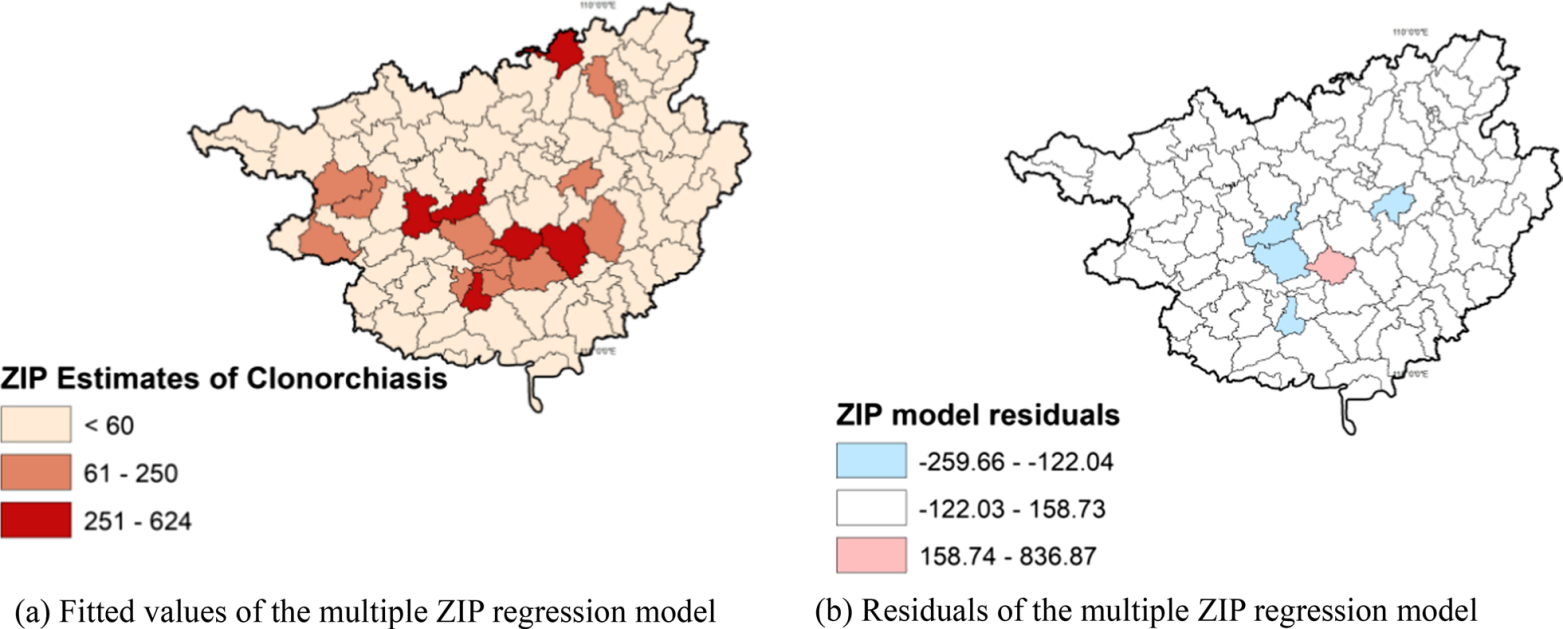
Maps depicting fitted values (**a**) and residuals (**b**) of the multiple Zero-inflated Poisson regression models. We drew this figure using Quantum GIS software

In the univariate ZIP regression model, the number of human clonorchiasis cases by county was significantly associated with all six variables (P-value < 0.05) (Table 2, Fig. 4, Additional file 1: Table S1). The variable named ‘Area’ indicate areas determined by a pre-existing regionalization of foodborne pathogens in Guangxi. However, considering the high correlation between these areas and the habit of raw fish consumption (correlation coefficient was 0.82 between the two categorical variables), this variable was removed from the multiple ZIP model. Among the meteorological factors that were significant in the simple regression model, given the high correlation between rainfall and highest temperature (0.49), highest temperature was removed from the final multivariable ZIP model (Table 2).

**Table 2:** Results of the cross-sectional analysis by fitting Zero-inflated Poisson (ZIP) regression models to human clonorchiasis reported in Guangxi from 2008 to 2017

Covariates	N	Simple ZIP regression		Multiple ZIP regression	
		Estimate^a^	S.E	Estimate^a^	S.E
Intercept				0.001 (0.001–0.002)*	0.17
*C. sinensis* Positive rate of fish samples	101	0.92 (0.91–0.93)*	0.51	0.92 (0.91–0.93)*	0.00
Raw fish consumption					
Type 1 (None)	33	Reference		Reference	
Type 2 (Seldom)	30	0.72 (0.60–0.837*	0.09	0.27 (0.22–0.34)*	0.11
Type 3 (Sometimes)	5	3.58 (2.96–4.32)*	0.09	0.60 (0.48–0.76)*	0.11
Type 4 (Frequent)	33	12.04 (10.51–13.72)*	0.09	5.52 (4.71–6.48)*	0.08
Area					
Non-endemic	8	Reference			
Light infection	37	4.99 (3.54–7.36)*	0.69		
Moderate infection	6	8.25 (5.73–12.31)*	0.52		
Heavy infection	16	17.12 (12.17–25.14)*	0.55		
Very heavy infection	13	36.20 (25.74–53.09)*	0.75		
Unknown	21	1.09 (0.72–1.70)	0.43		
Water area					
Rong River	10	Reference		Reference	
Liu River	11	0.38(0.35–0.42)*	0.04	0.90 (0.81–0.99)*	0.05
River along the South Coast of Guangxi	12	0.04 (0.03–0.05)*	0.09	0.15 (0.12–0.19)*	0.12
Changjiang River Valley	4	0.02 (0.01–0.04)*	0.30	0.34 (0.18–0.64)*	0.31
You River	13	0.51 (0.47–0.55)*	0.04	1.66 (1.46–1.87)*	0.06
Zuo River	6	0.10 (0.08–0.13)*	0.12	0.09 (0.07–0.11)*	0.12
Honghe River Basin	15	0.05 (0.04–0.06)*	0.11	0.05 (0.04–0.07)*	0.12
Qin River	2	0.09 (0.07–0.12)*	0.14	0.13 (0.09–0.17)*	0.15
Yu River	2	0.36 (0.32–0.41)*	0.05	0.18 (0.16–0.20)*	0.06
Xun River	10	0.04 (0.03–0.05)*	0.09	0.02 (0.01–0.02)*	0.10
He River	4	0.01 (0.00–0.02)*	0.28	0.12 (0.06–0.22)*	0.30
Gui River	12	0.10 (0.08–0.12)*	0.09	0.16 (0.13–0.19)*	0.10
Gross domestic product (yuan)					
≤ 31,127	50	Reference		Reference	
31,128–71,467	40	0.87 (0.81–0.93)*		0.25 (0.22–0.28)*	0.05
> 71,467	11	0.74 (0.67–0.82)*		0.18 (0.16–0.20)*	0.06
Environmental variables					
Evaporation	101	0.99 (0.99–0.99)*	0.51	0.97 (0.97–0.97)*	0.00
Rainfall	101	0.98 (0.98–0.99)*	0.51	1.00 (1.00–1.01)*	0.00
Highest temperature	101	1.60 (1.54–1.67)*	0.52		

Estimate^a^: Exponentiated values are presented and interpreted as ratios

N, Number of counties under each variable/category

^*^P-values from the Likelihood Ratio Test (< 0.05)

In the multiple ZIP regression model, counties with higher positive rates in fish samples had relatively few human cases reported compared to those that had low positive rates (0.92; with a narrow confidence interval indicating limited variation of the positive rates among the counties). When the counties were categorized based on their prominent raw fish consumption habits, compared to type 1 (i.e. no consumption of raw fish), counties with frequent consumption (type 4) had 5.52 times more cases of clonorchiasis. Compared to the counties in close proximity to the Rong river, counties around the You river had 1.66 times more cases. The number of cases was less in counties with a higher GDP (Fig. 3f). The average values of both rainfall and evaporation did not indicate a positive or negative association with human clonorchiasis.

The distribution of human clonorchiasis cases (i.e. the dependent variable) in Guangxi indicated a statistically significant trend of spatial autocorrelation between the counties (P-value 0.06, Fig. 4a). After the multiple ZIP model was fit, Moran's I test performed on the residuals of the model indicated no global spatial autocorrelation (P-value 0.97, Fig. 4b), suggesting that variables included in the regression model may have contributed to explaining the association and the similarities among the adjacent counties.

## Discussion

This study demonstrated that the infection of *C. sinensis* in freshwater fish was very common in Guangxi. Around two-thirds of counties in Guangxi detected the *C. sinensis* encysted metacercariae in local market/restaurant/fish pond samples. All counties except four had cases reported and the prevalence rate of clonorchiasis in Guangxi increased between 2008 and 2017. The detection of *C. sinensis* in humans and freshwater fish varied significantly by county. The prevalence of clonorchiasis at the county level was positively correlated with raw fish consumption habits and certain rivers. However, the positive rate of *C. sinensis* encysted metacercariae in fish samples was not positively correlated with human infections at the county level. This study may provide insights to the importance of fish species sampling in surveillance and emphasizes the importance of public health education on consuming raw fish dishes.

Consistent with Heilongjiang and Guangdong, other clonorchiasis prevalent provinces in China [12, 13], the infection of *C. sinensis* in *Pseudorasbora parva, Misgurnus anguillicaudatus* and some small fish were high in Guangxi. However, the total prevalence of *C. sinensis* in fish in Guangxi (9.7%) was lower than in Heilongjiang (20%) and Guangdong's Pearl River Delta (37.1%). But the infection rate of human clonorchiasis in Guangxi was higher than Heilongjiang in the 2003 national survey [7]. This may be due to the wide distribution of *C. sinensis* in the environment and the common raw fish consumption habits in Guangxi. Our study found that around two-thirds of counties in Guangxi detected the *C. sinensis* encysted metacercariae in local fish samples. Over 37% of counties had frequent or sometimes consumption of raw fish dishes as a home meal and more than 70% of counties could access raw fish dishes at local restaurants. As all fish species with scales can be used in making raw fish dishes in the cooking culture of Guangxi [31], people may become infected once they consume raw fish dishes, especially those containing fish species with high *C. sinensis* infection rates.

Clonorchiasis is on the rise in Guangxi, similar to the other areas in China [15, 32, 33]. This may due to easily access of raw fish dishes at restaurants and the opportunity of dining out increase with the economic development. More cases (58.9%) were reported between July and December may due to people often consume raw fish in summer (from May to October).The number of reported cases is mainly concentrated in the central areas that surround the capital city of Nanning. This was consistent with the raw fish consumption habits identified through the survey-based data collection at the county level [34, 35]. Clonorchiasis is known to be associated with the consumption of raw or undercooked fish [36, 37], and our study further confirms that the prevalence of clonorchiasis at the county level was positively correlated with the frequent raw fish consumption habit of local residents. However, it should be noted that the residents of around a third of counties in Guangxi did not consume any raw fish dishes. Worryingly, most of the counties (97/101) had at least one reported case. Consumption of raw fish is common throughout China [38] and a study in South Korea showed that certain village/communities in the region have a strong network of sharing raw fish dishes [39]. People from counties which had no or very little consumption may consume raw fish dishes from the other areas and become infected.

Our study showed that human clonorchiasis cases aggregated at the county level was not associated with per capita GDP or meteorological parameters including highest temperature, average evaporation, and average rainfall. If an association does exist, a more granular scale may be needed instead of aggregating at the county level. Moreover, obtaining average or highest values (with less variance between counties) to represent meteorological factors that vary over space and time may have undermined the contribution of these factors in the analysis. Other published studies reported that the prevalence of clonorchiasis were positively associated with temperature and rainfall, and negatively associated with relative humidity [6]. Out study showed that certain rivers were positively associated with human clonorchiasis cases at the county level. This was consistent with previous studies which found that the prevalence of *C. sinensis* infection varied among residents living in different rivers [8, 40]. In this study, the Rong river was chosen as the reference category given that it is the Northernmost branch of the Xi river system, and in agreement with a previous study [30], our study also shows that the majority of the Xi river system, especially the You river, was significantly associated with human clonorchiasis.

There are some limitations in this study. Clonorchiasis is not a notifiable disease in China, thus underreporting may have occurred. The number of reported cases in each county might be highly influenced by the awareness of local medical workers. Thus, there exists a gap between the reported cases and the estimated population under infection with *C. sinensis* in Guangxi. The increasing trend by year should be interpreted carefully, which could be attributed to the increasing trend, but also the strengthening in reporting. However, the information from reported cases still provided valuable information, especially the spatial distribution as demonstrated in this study. Secondly, the habit on consuming raw fish dishes was collected through a survey targeting local health officers or CDC staff. Clonorchiasis has a significantly different distribution by geography. Thus, there should also exist variations at the sub-county level. Future surveys at the individual level may provide more accurate data on consumption behaviors. For example, a survey-based study from Guangxi recently suggested that people continue to consume raw fish despite the knowledge on the risk because of the belief that anti-parasitic treatments are effective and the disease is mild [34]. Also, as there was no fish infection data in Guangxi previously, we used the fish data from 2016 to 2017 to correlate with human data between 2008 and 2017, the correlation may deviate with the real situation as the infection in fish may change over time.

## Conclusions

A high prevalence of *C. sinensis* infection was demonstrated in both humans and freshwater fish in Guangxi, China. Human clonorchiasis and *C. sinensis* infections in freshwater fish varied significantly by county, which could be attributed to the differences in environmental, social-economic, and behavioral determinants that are subjected to variations over time. The study highlights the importance of regular surveillance for both humans and freshwater fish to capture the changing trends. Concerted efforts in public education, prevention, and control need to be implemented in Guangxi, especially in highly endemic areas.

## Supplementary Information

**Additional file 1: Table S1.** AIC values of the model fits.

## Data Availability

The datasets used and/or analysed during the current study are available from the corresponding author on reasonable request.

## Abbreviations

*C. sinensis*: *C* *lonorchis sinensis*
ZIP: Zero-inflated Poisson regression
AIC: Akaike information criteria
GDP: Gross domestic product
OR: Odds ratios
CI: Confidence intervals
