# Supplementary material for: Epidemiological characterization of Clonorchis sinensis infection in humans and freshwater fish in Guangxi, China
Source: BMC Infect Dis. 2022 Mar 18;22:263. doi: 10.1186/s12879-022-07244-2 (PMC8932281; doi:10.1186/s12879-022-07244-2)
Supplement: Supplementary file 1 — Additional file 1: Table S1. AIC values of the model fits. [file 12879_2022_7244_MOESM1_ESM.docx]

# **Supplementary documents**

Supplement table 1. AIC values of the model fits.

|  | AIC values of simple ZIP model fits | AIC values of multiple ZIP model fits |
| --- | --- | --- |
| Rivers | 8177.71 |  |
| Raw fish consumption | 9199.94 |  |
| Areas | 10172.34 |  |
| Positive rate | 12571.59 |  |
| Rainfall | 13522.45 |  |
| Highest temperature | 13524.46 |  |
| Evaporation | 14176.94 |  |
| Gross domestic production (GDP) categories | 14165.61 |  |
| Full model with all 8 variables |  | 2981.36 |
| Reduced model with 7 variables  (i.e. full model without areas) |  | 3746.32 |
| **Reduced model with 6 variables (i.e. Reduced model without highest temperature)** |  | **3035.106*** |
| Reduced model without GDP |  | 4153.49 |
| Reduced model without GDP and highest temperature |  | 4161.60 |
| Reduced model without HT2016 and river |  | 7785.13 |

*Finalized multiple ZIP model
